# Toward an Interactive Reinforcement Based Learning Framework for Human Robot Collaborative Assembly Processes

**DOI:** 10.3389/frobt.2018.00126

**Published:** 2018-11-22

**Authors:** Sharath Chandra Akkaladevi, Matthias Plasch, Sriniwas Maddukuri, Christian Eitzinger, Andreas Pichler, Bernhard Rinner

**Affiliations:** ^1^Profactor GmbH, Steyr-Gleink, Steyr, Austria; ^2^Institute of Networked and Embedded Systems, Alpen-Adria-Universität Klagenfurt, Klagenfurt, Austria

**Keywords:** human robot collaboration, interactive reinforcement learning, reasoning, knowledge modeling, cognition

## Abstract

As manufacturing demographics change from mass production to mass customization, advances in human-robot interaction in industries have taken many forms. However, the topic of reducing the programming effort required by an expert using natural modes of communication is still open. To answer this challenge, we propose an approach based on Interactive Reinforcement Learning that learns a complete collaborative assembly process. The learning approach is done in two steps. First step consists of modeling simple tasks that compose the assembly process, using task based formalism. The robotic system then uses these modeled simple tasks and proposes to the user a set of possible actions at each step of the assembly process via a GUI. The user then “interacts” with the robotic system by selecting an option from the given choice. The robot records the action chosen and performs it, progressing the assembly process. Thereby, the user teaches the system which task to perform when. In order to reduce the number of actions proposed, the system considers additional information such as user and robot capabilities and object affordances. These set of action proposals are further reduced by modeling the proposed actions into a goal based hierarchy and by including action prerequisites. The learning framework highlights its ability to learn a complicated human robot collaborative assembly process in a user intuitive fashion. The framework also allows different users to teach different assembly processes to the robot.

## Introduction

**Figure 1:**
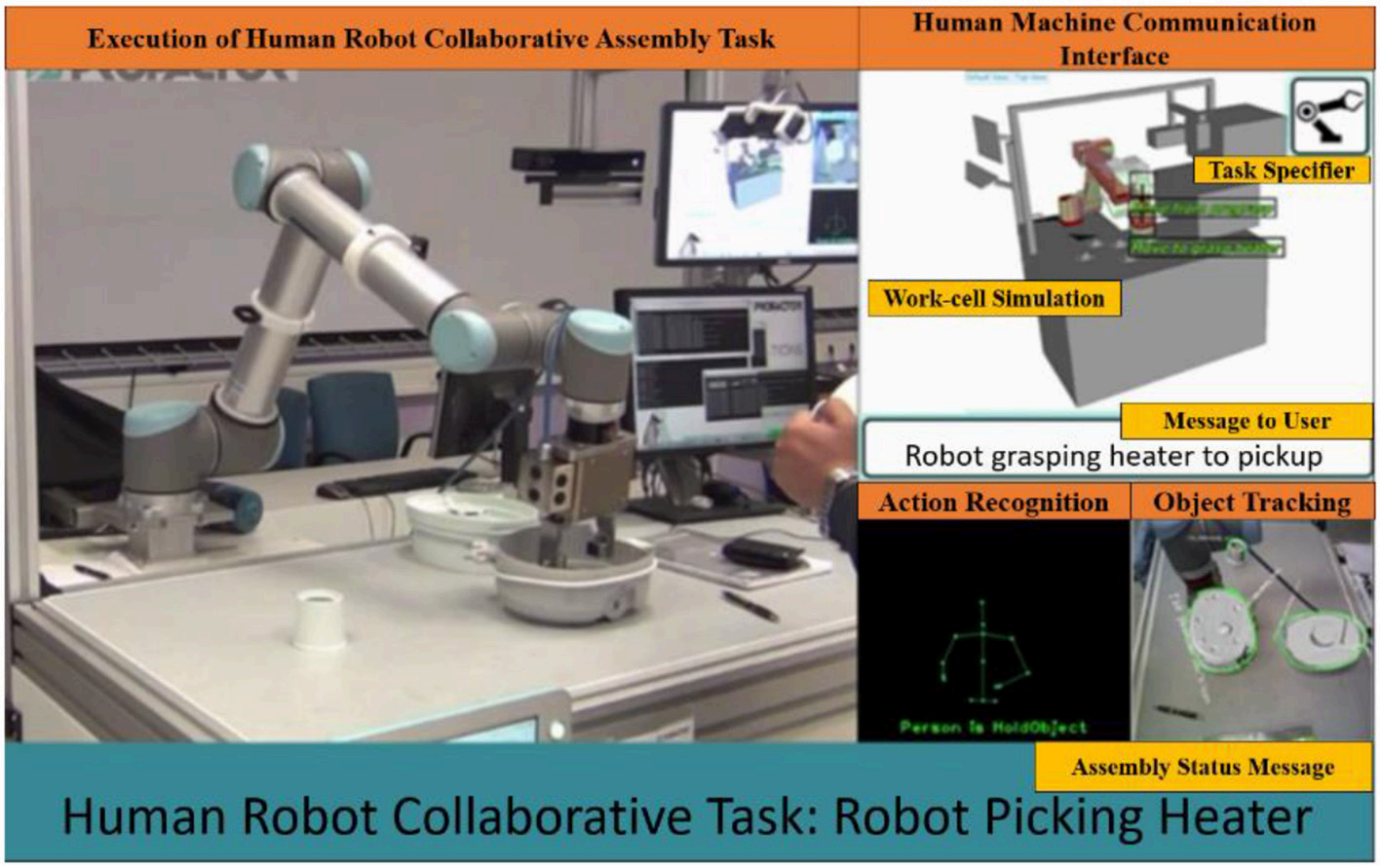
Robot manipulating object of interest in coordination with human user in an integrated cognitive architecture, where the robot perceives, reasons, plans, executes and adapts. The image also depicts the object tracking, action recognition and communication Akkaladevi et al. (2018).

Human robot interaction (HRI) is realized in various forms. Depending on the kind of interaction and the nature of the task involved, different classifications of HRI exist in literature (Shen, 2015). In this work, we use the classification of HRI in industrial scenarios as given in Pichler et al. (2017); they are (a) **Human robot coexistence**—where both agents (human and robot) operate in a close proximity on different tasks (b) **Human robot assistance**—where the robot passively aids the human in a task (helping in lifting heavy objects). (c) **Human robot collaboration**—where both agents simultaneously work on the same work piece (each agent has their own task to do on the work piece). An example of such a collaborative robotic system is shown in Figure 1.

In this work, we present such a human robot collaborative architecture. The main focus of the work is on the learning framework that is based on this cognitive architecture, that enables a user to easily teach the robotic system a complete human robot collaborative process. To achieve this, the work combines two different kinds of learning methodologies. Firstly, the robotic system uses task based formalism (Nicolescu and Mataric, 2003), to learn simple manipulation tasks (such as picking, placing, etc) of objects. Then these simple tasks are combined in an intuitive fashion to learn the complete assembly process. Where, given a set of objects, the robotic system having learned simple tasks, proposes a set of possibilities to the user. The user then selects the appropriate task to be carried out at that point of time. These set of task proposals are done in an intelligent fashion considering the user capabilities, robot capabilities and manipulations that are possible on the available objects. This interaction process of robot proposal and user interactions is based on interactive reinforcement learning that is described in detail in the next sections.

The work presented in this paper is based on our extensive previous work (Akkaladevi et al., 2016b, 2017b,c, 2018; Pichler et al., 2017). The main contribution of this paper can be summarized as follows: First, we present an interactive reinforcement based learning framework, where the robotic system proactively proposes a set of solutions (in an intelligent fashion) to users and learns the assembly process. Second, we improve the knowledge-modeling framework to easily represent human robot collaborative processes. Finally, we clarify the role of the action hierarchy and action prerequisites in reducing the effort required in learning the assembly process. Note that the discussion of related work in this paper represents a revised version of the discussions in Pichler et al. (2017) and Akkaladevi et al. (2017b) with additional details.

The remaining part of the paper is organized as follows. The state of the art dealing with various learning approaches, where a user teaches a robot is provided in detail in section State of the Art. The architecture that enables the proposed learning approach is given in section KoMoCog Architecture. The main idea of the learning approach and the way in which simple tasks are learned (using task based formalism) and then later combined to form the complete assembly process (user interaction based reinforcement learning) is given in section Learning Methodology. The experimental setup and the complete learning process in action are shown and discussed in section Experimental Setup and the Learning approach in Practice. Finally, some concluding remarks and possible next steps are given in section Evaluation.

## State of the art

In the research community there is a growing interest to solve the challenge of robots learning in complex real-world environments (Dautenhahn, 2007; Goodrich and Schultz, 2007; Bauer et al., 2008; Argall et al., 2009). In the literature, different methods for developing agents, which can learn activities from a human instructor, are described. Common to all methods is the reduction of programming effort. However, the problem of reducing the programming effort required by an expert using natural modes of communication is still an open question (Pedersen et al., 2016). When viewed from a broader perspective, learning approaches can be classified as follows:

- Learning by advice
- Learning by programming
- Learning by demonstration
- Learning by interaction

**Learning by advice:** This is made possible by the use of natural forms of communication. The authors in Maclin and Shavlik (1996) developed a method whereby advice is given to the learning agent (Reinforcement Learning). In the context of Markov decision-processes (MDPs), advice means that the agent is suggested to execute an action if a particular condition applies. The use of Natural-Language to communicate this advice is particularly effective for non-experts. Such an interface was implemented by Kuhlmann et al. (2004) and Moreno et al. (2004). The detection of Natural-Language data has not yet been solved. Therefore, many approaches require that advice be coded in the syntax of scripting or programming languages. This makes access for non-experts more difficult.

**Learning by programming:** The approaches falling under this category use the task-level programming paradigm for easy and quick re-programming by non-experts. The task-level programming paradigm (Nicolescu and Mataric, 2003) is built upon on a set of *actions*, where the *actions* have the capability to alter the current world state. These set of *actions* can be seen as formal descriptions of compliant robot motions and are composed of *primitives*. These *primitives* are simple, atomic movements, and are combined to form a task (Finkemeyer et al., 2005). The task could consists of a single *action* in simple cases and have multitude of *actions* in complex scenarios. An example of a *primitive* is typically a sensory input or a single robot motion, described using the Task Frame Formalism (TFF) (Bruyninckx and De Schutter, 1996). From the perspective of the robot operation, any given assembly task is (and could be) broken down into a form of *action primitives* which the robot can interpret (Mosemann and Wahl, 2001). The main prerequisite for using a (TFF) is to model the world state (in terms of action primitives) and maintain it online. The main drawback of this approach is that the modeling complexity increases exponentially as the complexity of the task increases. To alleviate this problem, we suggest using generic *recipes* (Nicolescu and Mataric, 2003) instead of using such *action primitives* to build up the assembly task. The terms *recipe* and skill are used analogously in this paper. When compared to the *action primitives*, the *recipes* are abstracted on a higher level and hence form a bridge between complex tasks and the *primitives*.

There is an active interest in the research community to use TFF and the approaches differ based on how *primitives* are combined to describe the task. For example, to support hierarchies and concurrencies in the task, a visual programming tool for defining the flow control is proposed in Steinmetz and Weitschat (2016). In Pedersen and Krüger (2015), an automated task planning and execution system is shown as a sequence of skills and their parameters, based on the desired goal state and the current state from the world model. To deal with variations in the assembly process, a programming framework that uses knowledge- based components is proposed in Dean-Leon et al. (2016). To allow portability across different platforms, the work in Holz et al. (2015) details an integrated skill-based framework coupled with task planning.

**Learning by demonstration (LBD):** Is a popular method by which agents learn by physically presenting the task through the human being Billard et al. (2008). The work in Argall et al. (2009) provides a detailed overview of relevant methods. A disadvantage of LBD is that complicated tasks can only be presented with difficulty (e.g., if several robotic systems are to solve a problem at the same time). A human-in-the-loop adaptation to correct a batch-learned policy iteratively and improve accuracy and precision is described in Ko et al. (2015). To allow users to generate skills and robot program primitives for later refinement and re-use is proposed in Stenmark and Topp (2016).

**Learning by interaction:** With the environment is another interesting approach. Recent trend points toward applying Reinforcement learning (RL) (Sutton and Barto, 1998) for this purpose. In these approaches, learning arises from interaction with the environment. The agent (robot) learns how to behave (which actions to perform when) in order to complete a task in the given environment. The agent learning process (using RL) takes place over discrete time steps by interacting with the environment and gaining experience about the outcome. To reach an optimal policy (the set of actions that lead to the maximum reward), a substantial interaction with the environment is required. As a result, the RL approach leads to a memory-intensive storage of all state action pairs in case of complex tasks (Kartoun et al., 2010). Slow convergence toward a satisfactory solution is also another drawback of the RL approaches. To overcome these problems, recent approaches use a human teacher in the loop to provide feedback (reward) instead of allowing the agent to “aimlessly” interact with the environment (Thomaz et al., 2006; Suay and Chernova, 2011; Griffith et al., 2013; Knox et al., 2013). The human teacher in the loop provides feedback, which is used by RL approach to speed up the convergence time required to reach an optimal policy. Such approaches are termed as interactive reinforcement learning (IRL) approaches.

The work in Thomaz et al. (2006) argues that a run-time human feedback as a reward to the IRL approach is beneficial for both the human teacher (to understand the perspective of the robot) and the robot (which learns the optimal policy with help of the learning algorithm). The user study in this work suggests that the human teachers employ the feedback via a single communication channel for various communicative intents (feedback, guidance, and motivation). Using this as basis, the work in Suay and Chernova (2011) conducted a study to realize such IRL approach for efficient real-world robotic systems. In this study, the feedback provided by the user is 2-fold: first a reward for preceding actions of the robot and then a guidance for subsequent actions. The results show that such an approach reduces the learning time of the robot in particular when a large state-space (number of interactions possible by the robot in a given environment) is considered. Another approach to accelerate the learning process is given in Peng et al. (2016).

To train an agent manually via evaluate reinforcement, a framework that uses real-valued feedback from a human trainer about the agent behavior is described in Knox and Stone (2009). Such a feedback allows the trainer to build the agent's policy (interactive shaping). This shaping directly modifies the action policy (the selection mechanism) of the IRL algorithm. Instead of using the feedback as an indirect influence, the work in Griffith et al. (2013) uses it to make a direct statement about the action policy. A framework (TAMER) to apply the approach mentioned in Knox and Stone (2009) for real-world robotic system is given in Knox et al. (2013). In Rozo et al. (2013, 2014), the robot learns both the desired path and the required amount of force to apply on an object during the interaction.

There are not many approaches which attempt applying IRL toward real-world scenarios. This is especially true for robot learning in real-world industrial settings. The work in Pedersen et al. (2016) and Pedersen and Krüger (2015) demonstrates partial application of IRL to practical demonstrations. Their work demonstrates a use case specific learning and are limited to the number of actual interactions between the human and the robot. Also, a complete human robot collaborative assembly process with varying number of objects is not considered.

In contrast, our work builds upon the idea of task based programming (Pichler et al., 2017) to teach the robot simple tasks and then uses reinforcement based interactive learning to teach the robot the sequence of these simple tasks that correspond to a complete assembly process. The advantage of the proposed approach is that the robotic system (that has already learned simple tasks) provides a set of solutions to the user for a given set of objects, the user then selects the optimal sequence of these tasks to complete the assembly process.

The contributions to the state of the art include:

- Easy intuitive programming framework to learn an assembly process using user interactions
- Applicability to human robot collaborative assembly tasks with varying complexity.

## KoMoCog architecture

**Figure 2:**
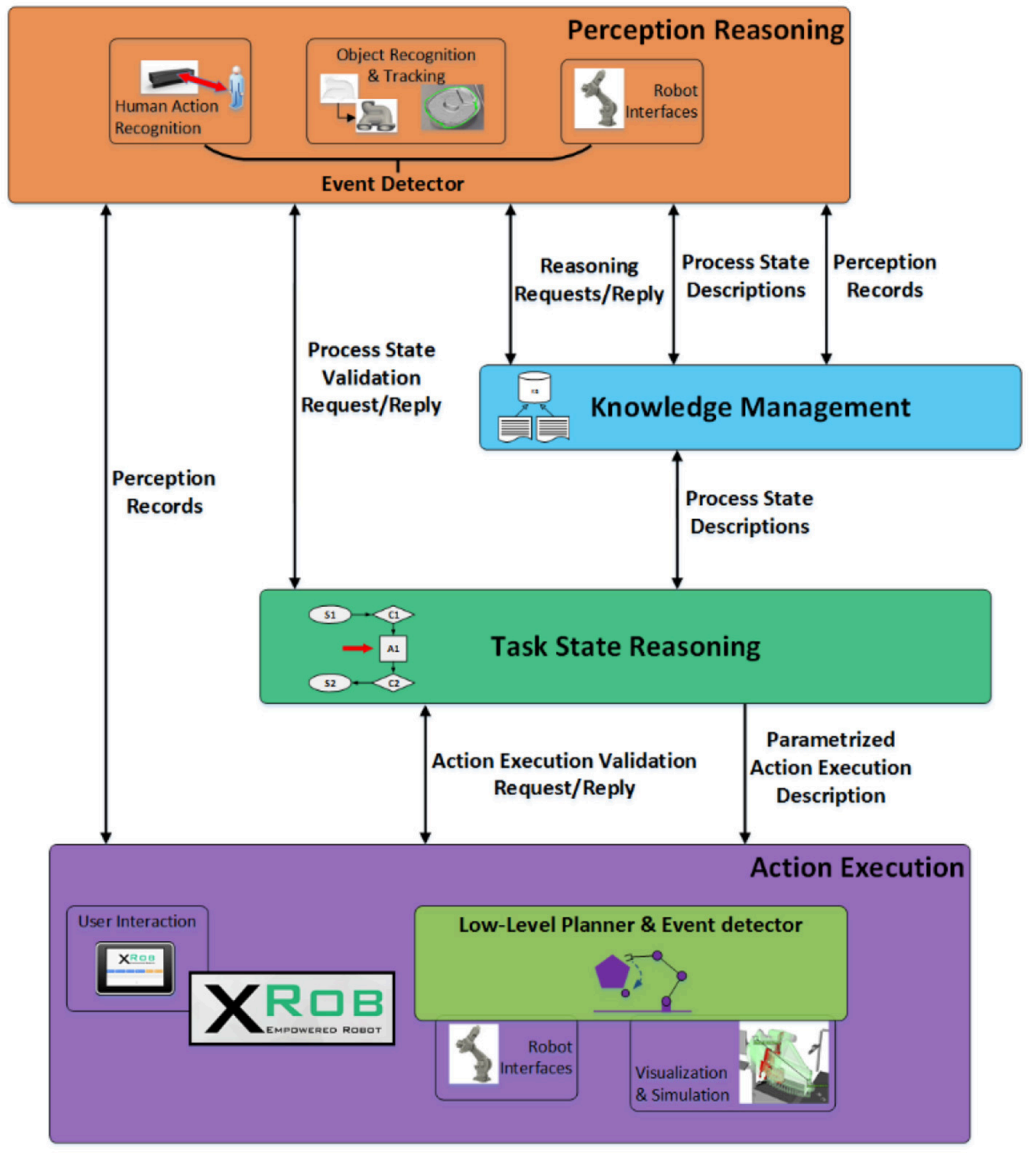
KoMoCog Architecture for human robot collaboration depicting various modules and their communication interfaces.

To deal with the HRC assembly process the robotic system should be enabled with cognitive capabilities in order to perceive, reason, plan and execute as shown in Figure 2.

The learning framework is based on the KoMoCog architecture and is equipped with the following: (a) Perception Reasoning (PR) understands and interprets the current state of the environment and the assembly task from data provided by the perception system combined with the robot state received from the planning and execution system. The PR is equipped with real-time 3D tracking of objects with the help of the Multi Object Tracker (Akkaladevi et al., 2016a, 2017a) and an action recognition system to recognize the current action performed by the human (Akkaladevi and Heindl, 2015). The object tracker and the action recognition system are combined to recognize the events (actions that interact with objects) using an event detector (Akkaladevi et al., 2016b); (b) Knowledge Management (KM) represents and abstracts different aspects of the assembly process in the HRC environment. This includes representing the abilities and possible activities of the human and the robot, interplay between human/robot activities and object configurations, current state of the human and the robot with respect to the task; (c) Task State Reasoning (TSR) reasons about the current assembly state by combining information about the current state (given by perception reasoning) and the assembly process knowledge (knowledge management), to make decisions, to plan the next actions accordingly in coordination with the planning and execution system, to behave intelligently, to interact naturally with humans and aid in completing the task. The knowledge management, perception reasoning and the task state reasoning modules together constitute the Cognitive Reasoning System in the architecture. (d) Action Execution (AE): This module is responsible for plan generation, where the plan consists of actions required to achieve the given task (goals). This includes planning of the task, scheduling of the actions and also includes planning under uncertainty for an efficient human robot collaboration. The plans generated are carried out in real-world where the robot plans its path (path/navigation planning) and manipulates the environment accordingly. The AE is also equipped with communication interfaces that provide a GUI- interface for human robot communication and simulations of the robot's planned behavior. A detailed description of the architecture is given in Akkaladevi et al. (2016b) and Pichler et al. (2017).

### Modeling knowledge in HRC assembly process

An assembly process *AP* in its simplest form can be defined as a sequence of States **S**, a set of Events **V** and a set of Relations **R**. The set of States **S** defines the individual steps of the assembly process. The set of Events **V** drives the progress of the assembly process from one step to another. The Relations **R** specify the effect of a given Event **V** on a given State **S** in progressing the assembly process. The architecture consists of **KM** that defines the corresponding data structures to manage and abstract the *knowledge of the assembly process*. This includes task state descriptions, robotic system configuration, capabilities of the robotic system and human operator, involved objects, their configurations and affordances, the properties of agent's (human, robot) actions and their corresponding effects on objects. The KM framework is an extended implementation of KnowRob (Tenorth and Beetz, 2013), as KnowRob provides the following knowledge processing features: (a) mechanisms and tools for action centric representation, (b) automated acquisition of grounded concepts through observation and experience, (c) reasoning about and managing uncertainty, and fast inference.

The knowledge is represented using ontologies (description logics) based on the Web Ontology Language (OWL). SWI Prolog is used for loading, accessing and querying the ontologies. The representation consists of two levels: **Classes** that abstract terminological knowledge (type of objects, events and actions) and **Instances** which represent the actual physical objects or the actions that are actually performed. **Properties** establish relations (links) between Classes, and these links are also valid for the Instances of the respective Classes. For example, Properties define if an Agent *Agent ϵ* {*Human, Robot*} can perform a particular action (defined in Classes) on/with a *Target ϵ* {*Objects, Robot, Human*}.

The KnowRob framework provides a suitable basis (base ontologies) for modeling actions, objects of interest, and capabilities of humans and robots. A collection of Prolog rules are also provided for parsing ontologies and loading them into the Prolog database, thus making the ontology data accessible for database queries. We extended the base ontologies to express an HRC Assembly Process description. Moreover, Prolog rules were also extended to provide functionalities such as (a) posting a snapshot created by the Perception System into the database, (b) checking if recorded perception data fulfills the constraints of an assembly process state, (c) projecting the expected outcome of an action that is planned for execution, and (d) deriving the expected succeeding assembly process state. All these functions rely on Prolog queries (e.g., unification and proof search in the database, difference-list operations) and ontologies (e.g., deducing facts which are not explicitely asserted in a database through so-called “computables” (Tenorth and Beetz, 2013 and ontology-reasoning).

An AP in HRC involves presence and manipulation of several objects. The AP consists of different steps, where each step requires a particular kind of manipulation on specific objects. For the robotic system to successfully complete the AP, it should (a) determine the current state in the AP, (b) choose/plan a necessary action to progress the AP, (c) execute the planned action, (d) and verify if the action was successful. All these steps are iteratively executed until the AP is successfully completed.

#### Determine the current state in the AP

Given the assembly process and assuming it begins with the initial state, the TSR queries the knowledge management system for information regarding the current state (initial state) in the AP. This data includes process state constraints that describe (a) spatial relations between objects, present in the workspace, (b) required states of the human and robot and (c) Event descriptions that lead to subsequent assembly process states. Based on the given spatial relation constraints the TSR deduces the objects of interest for the given state. A snapshot of the current scene (object locations—Tracking, human and robot states—Action Recognition and Robot Proprioception) needs to be created. The TSR triggers the PR with the given information (a) objects of interest available in workspace and b) robot and human in given state (e.g., IDLE state), and requests the validation of these constraints. The PR then waits for a stable response of all perception sources, i.e., Object Tracker, Action Recognition and Robot proprioceptive feedback to validate the information provided by TSR. Afterwards, PR posts this snapshot into the Prolog database using a specific rule. Now the verification rule is triggered, which uses the KnowRob built-in computable *comp_spatial* to verify if the spatial relation constraints are fulfilled and also compares detected human and robot states with the given process state constraints. The built-in computable are functions that help to verify the spatial relations of objects, given the current configuration of objects in the AP. If the verification succeeds, the given process state is assumed to be verified and the related Event descriptions are evaluated to deduce the next action to be executed.

The given Cognitive Architecture needs to deal with multiple instances of the same object type in the assembly process. In order to express a spatial relation constraint to be valid for a number of instances, we combined the expressiveness of OWL-Classes and their related OWL-Instances: We model a certain spatial relation of a certain pair of object types as an OWL-Class (e.g., “Sphere-onTopOf-WorkTable”). To express a number of distinct configurations of this spatial relation, an instance of the considered OWL-Class is created and asserted with an OWL-Data-Property (e.g., an integer value) that describes the required quantity.

#### Planning of actions

The physical execution of an action, deduced by the TSR, requires its proper parametrization based on the given assembly process knowledge. Each type of action is modeled as an OWL-Class in the assembly process specific ontology. An action type OWL-Class describes the principal primitive (e.g., *PickAndHold* or *Insert*) and also describes the object types and targets affected by that action, as well as the type of actor that is capable of executing it. The related parametrization problem is described as finding concrete instances of objects, targets and the actor and generating a specific action instance. Considering the fact of multiple object instances available, e.g., for picking an object “Sphere,” the main question is the following: Which object instance shall be chosen? Our solution to this problem is to let the Planning and Execution System (PES) decide on selecting an appropriate instance. Even before triggering the execution, the TSR computes the expected action outcome, by projecting the potential action result with respect to the current state in order to acquire information on the expected changes in the environment (added/removed number of object instances, changed states of human and robot).

#### Execution of planned action

For triggering the execution, TSR executes a Prolog rule to get all possible candidates for object instances, target instances, and actor instances available. This data is forwarded to the PES that triggers human action execution (notification on GUI) or robot execution, dependent on the actor type, and configures the PR accordingly to initiate verification of the action.

The Verification of action executed is performed in two steps. Firstly, the response of the PES (i.e., success or failure) is considered and second, the TSR tries to verify the whether the expected changes in environment did happen accordingly (e.g., object instances removed/added). This is performed by configuring the PR to check whether specific objects were removed/added at certain locations, or a human/robot state change has happened. If the action results could be verified, the TSR tries to compare the predicted state (using the given assembly process knowledge) to the perceived current state. From this step on, the procedure is repeated until a final state is reached.

## Learning methodology

A detailed description of the task based formalism to reduce the programming effort for users is given in Pichler et al. (2017). However, this approach is suitable for teaching a task that has lower complexity (does not consists of too many human robot object interactions). It is possible to teach a complicated task using the approach mentioned in Pichler et al. (2017), but the effort required is exponentially increasing depending on the complexity. The mathematical description of the complex assembly process is given in section Formal Description of Assembly Process.

The solution to teach the robotic system a complex human robot collaborative assembly process is to first teach the simple tasks (that include one or two agent-object interactions). During the second stage, the robotic system can propose a set of possible simple tasks (actions). The reason for choosing the robot to propose a set of actions is to make the learning process more interactive and flexible. The user can then choose the sequence in which these actions should take place, thereby creating a recipe for the complete assembly process. However, simply proposing a set of actions to the user (given the already learned simple tasks) is not a valid solution, as there could be many possible actions and as a result could overwhelm the user with too many options to choose from. For example, consider the following situation, as shown in Table 1. Let an action be performed by either the robot or the human. In such a scenario, given 7 Objects and (5 + 1) actions each for the robot and the human, the total number of possible actions proposed would be (7 ^*^ 5) +1 for each robot and the human. **Resulting in a total of 72 actions**. Such a huge state-action space (possible actions for the current state) for proposing actions is too much for users to handle.

**Table 1:** An example use case with type of object and action possibilities.

**Objects**	**Possible actions**
Base (B)	Pick
Heater (H)	Hold
Ring (R)	Mount
Tray (T)	Receive
Compound (H+B)	Place
Compound (H+B+R)	Idle
Compound (H+B+R+T)

To reduce the state-action space and enable fluent human robot collaboration we propose the following algorithm as shown in Algorithm 1. We assume that the assembly process starts with an initially known state (known object, human, robot and object configuration in the environment). The system first detects the available objects, the current human state and robot state (as mentioned in section KoMoCog Architecture) and proposes a set of possible actions. The initially generated possibilities are reduced in two steps. First, the user capabilities and the robot capabilities together with object affordances are considered to reduce the action possibilities. Then, these action possibilities are further reduced using action hierarchy and action prerequisites. More details are described in sections Modeling of Human Capabilities, Robot Capabilities, and Object Affordances and Action Hierarchy and Prerequisites, respectively. Given a reduced list of action possibilities, the user is requested to select a suitable action. Once selected, the action is executed (in case the human chooses to perform the action, the event detection system waits until the action is executed) and then the resulting state is recorded. The user is also to notify the system if the resulting state is a final state or not. In case of a final state, the assembly process is completed and the complete sequence is stored. Otherwise, the system continues with proposing a next set of possible action in the resulting state.

**Algorithm 1:**
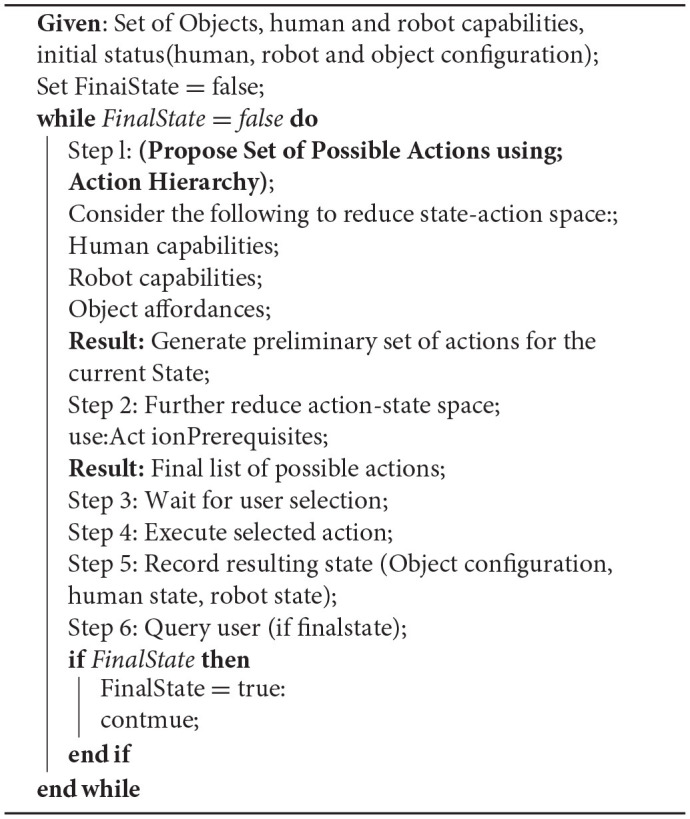
Algorithm describing the learning process.

### Formal description of the assembly process

An assembly process (AP) in its simplest form can be defined as a sequence of States *S*, a set of Events *V* and a set of Relations *R*. The terms State and task state are used analogously in this work. The set of States *S* defines the individual steps of the assembly process. The set of Events *V* drives the progress of the assembly process from one state to another. The Relations *R* specify the effect of a given Event *V* on a given State *S* in progressing the assembly process. A detailed formal description of the *AP* and its constituents is given in Akkaladevi et al. (2016b). The architecture consists of a knowledge management framework based on KnowRob (Tenorth and Beetz, 2013), that defines the corresponding data structures to manage and abstract the knowledge of the assembly process. This includes task state descriptions, robotic system configuration, capabilities of the robotic system and human, involved objects, their configurations and affordances, the properties of agent's (human, robot) actions and their corresponding effects on objects. The advantage of using KnowRob framework as basis is that it provides the knowledge processing features that include: (a) tools and mechanisms for action-centric representation (b) automated acquisition of grounded concepts through observation and experience (c) fast inference and reasoning capabilities with possibility to manage uncertainties. Ontologies (description logics) based on the Web Ontology Language (OWL) is used for knowledge representation. The SWI Prolog engine is used for loading, accessing‘ and querying the ontologies. The knowledge representation consists of two levels: Classes that abstract terminological knowledge (type of objects, events and actions—taxonomic fashion) and Instances which represent the actual physical objects or the actions that are actually performed. Properties establish relations (links) between Classes, and these links are also valid for the Instances of the respective Classes. For example, Properties define if an *Agent* ϵ *{Human, Robot}* can perform a particular action (defined in Classes) on/with a *Target* ϵ *{Objects, Robot, Human}* (Akkaladevi et al., 2016b, 2017b).

### Modeling of human capabilities, robot capabilities, and object affordances

The term capability refers to the possibility of an agent (human, robot or both) to perform a particular action on a particular object. The term object affordances in this context means, the possible actions that an object provides for an agent.

**Figure 3:**
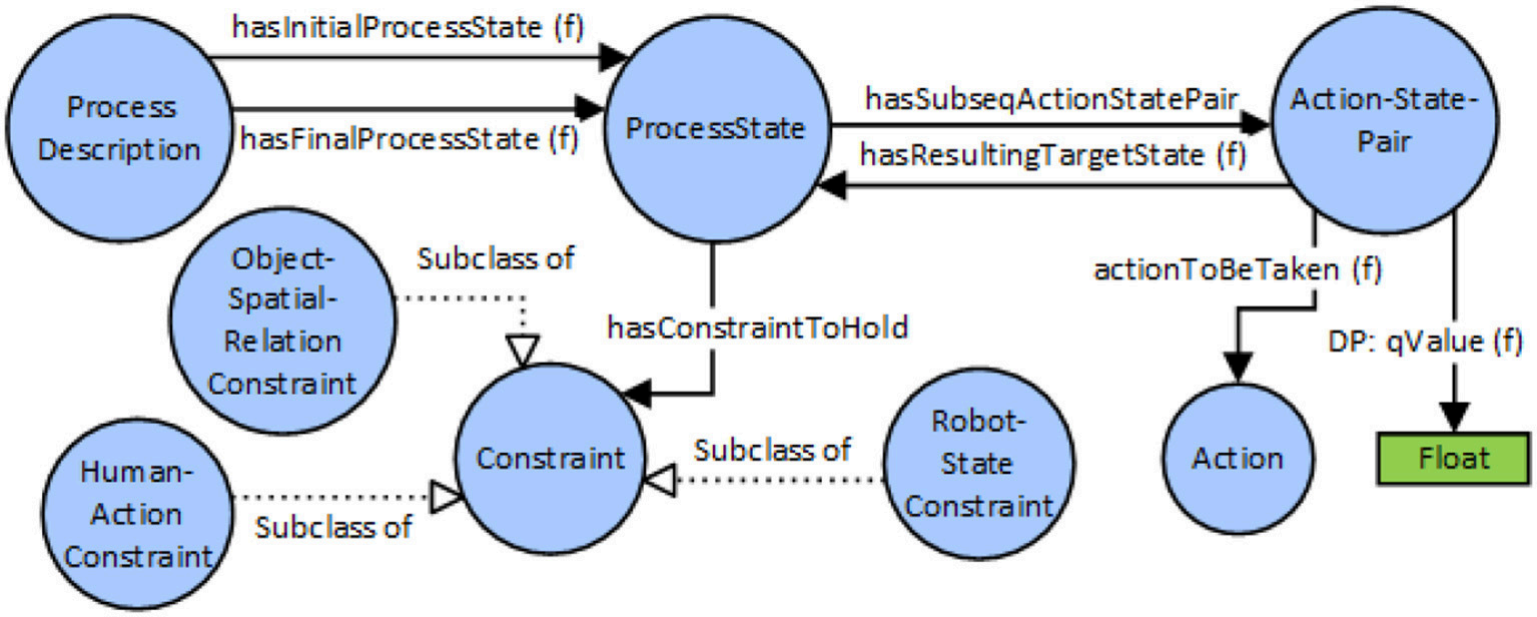
Schematic overview of knowledge representation for the assembly process.

A simplified, schematic overview of the knowledge representation for Assembly Process model is given in Figure 3. Blue circles represent classes (also called concepts) in OWL. Arrows describe relations of different types. Object-Properties describe relations among classes, and apply for all instances of a class too. Data-Properties are used to relate literals of simple data types with classes and instances. The ‘SubclassOf' property is used to establish class hierarchies, where a sub-class inherits property restrictions from its super-class.

A ProcessDescription refers to exactly one initial and one final ProcessState (PS). Starting from the initial PS, subsequent PSs can be reached via Action-State-Pairs (APSs). An ASP denotes a possible transition from a given PS to a subsequent PS, which will be taken if the Action associated to the ASP was executed. By associating more than one ASP with a PS, different execution paths or variants for an assembly process can be modeled. If more than one ASP is existing for a certain PS, each ASP needs to be associated with a literal Q-Value in order to provide a specific weightage factor for a certain ASP transition.

A ProcessState is characterized by an arbitrary number of constraints, currently three different types: Object-Spatial-RelationConstraints describe a spatial relationship between two object instances (eg: onTopOf, toTheSideOf, inFrontOf,…). HumanActionConstraints describe an action of the human actor, optionally applied to an object (eg Idle, Holding-Object-X, Picking-Object-Y,…). Robot-StateConstraints describe a required state of the robot system (e.g.: Idle, Reached-Target X,…). This description of a PS serves as basis for creating hypotheses on the current state of an assembly process, considering the current state of the environment.

**Figure 4:**
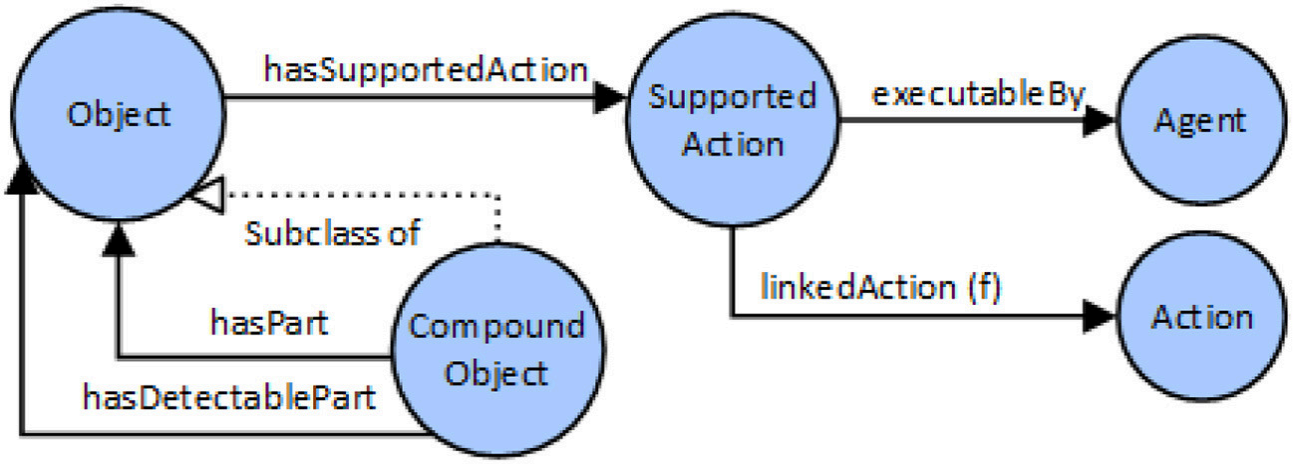
Modeling of object affordances and compound objects.

Figure 4 describes the definition of objects and compound objects, as well as modeling of object-affordances; i.e., actions that can be executed on a specific object type and is that is therefore “supported” by the object. A Compound Object consists of part-objects, where certain sub-parts of the compound object can also be declared as visually detectable. Visually detectable means that the object part is visible from outside, and there is a change to enable detection using an object recognition system.

For each object class, a list of actions can be defined that are supported by the concrete object. For example, if object class “XY” can be picked by a human (considering e.g., weight of the object and shape), an Instance of “SupportedAction” class is created and linked to the class of “Human” agent and to the action class “PickObject.”

As a result, with the help of modeled human and robot capabilities and object affordances the possible set of action proposals can be reduced. Considering the earlier example in Table 1, we can reduce the proposal of actions to the one given in Table 2.

**Table 2:** The state-action space after considering user capabilities, robot capabilities and object affordances.

**Objects of interest**	**Human capabilities (Hu)**	**Robot capabilities (Ro)**
**PRIOR KNOWLEDGE**		
Base (B)	Pick 3	Pick 4
Heater (H)	Hold 3	Show 4
Ring (R)	Mount 3	Handover 4
Tray (T)	Receive 3	Place 4
Compound (H + B)	Place 3
Compound (H + B + R)	Idle 1	Idle 1
Compound (H + B + R + T)	

*Total, 7 Objects; 16 Human actions + 17 Robot actions = 33 actions*.

In Table 2, the use case is modeled in such a fashion that the user cannot manipulate object (heater), while the robot cannot manipulate object (base). Given this information, the number of possible actions are reduced drastically **from 72 action to 33 actions** as shown in Table 2. However, 33 possible actions are still too many to be considered for user interaction and hence they need to be further reduced. This is done by dividing the possible actions into a hierarchy and including prerequisites as described in the next section.

### Action hierarchy and prerequisites

The list of action possibilities given in Table 1 are categorized into three layers as shown in Table 3.

**Table 3:** Hierarchy of action possibilities.

**Atomic actions**	**Basic actions**	**Collaborative actions**
**Single motion single actor**	**Multiple motions single actor**	**Multiple motions multiple actors**
Pick object(Human or robot)	Pick and place: Pick object + Place object on location	Mount: Robot idle + Human insert object onto other object
Place object on location (Human or robot)	Pick and insert: Pick object + Insert object onto other object	Pick and mount: Robot idle + Human pick and insert object onto other object
Insert object onto other object (Human or robot)	Pick and hold: Pick object + Hold object	Handover: Robot idle+ Human receive object+ Robot release object+ Robot go back
Hold object (Human or robot)
Idle (Human or robot)
Receive object (Human)
Release (Robot)
Move to position (Robot)

As shown in Table 3, **Atomic Actions** are generic, fundamental movements and are applicable to the representation of actions performed by humans or robots, e.g., “Pick object.” Atomic actions are independent of the concrete assembling process and represent the basic building blocks for more complex actions. They are adapted for a specific assembly task by means of a parameter assignment, but they do not usually lead to the assembly process progressing from one state to the next. **Basic Actions** are specific to the robot or humans and consist of a combination of atomic actions (e.g., “pick and hold object”). A basic action is always associated with prerequisites, which determine the feasibility of the action. The preconditions allow a logical linking of the basic actions to collaborative actions, which represent the highest hierarchy level. **Collaborative actions** are always a combination of actions involving both actors, humans and the robot (“handover of an object”). Basic and Collaborative Actions usually lead from one assembly process state to the next.

**Figure 5:**
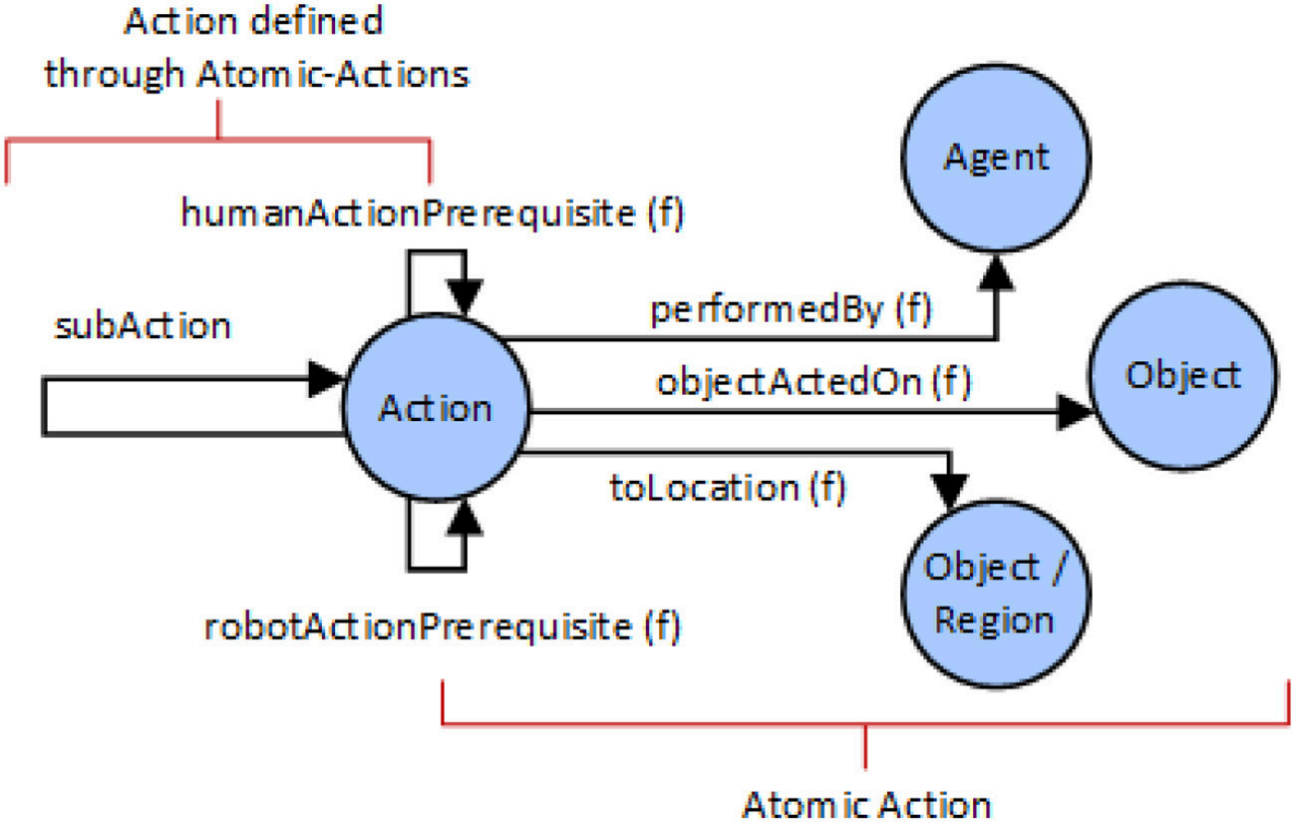
Semantic modeling of actions.

For the description of atomic actions, the following assumption was made: An Atomic Action is executed by exactly one agent (property performedBy), can involve at most one object of interest (objectActedOn) and can be applied at a certain location or to a certain object (toLocation). The capability of an agent to be able to perform a certain class of action, independent of the involved object types, is also given by the performedBy property. For example, if property performedBy restricts a certain action class to values of “Human or Robot,” both types of actors Robot and Human can perform the action. The semantic modeling of these actions is given in Figure 5. Note that only basic actions and collaborative actions are then proposed as they are goal oriented. The atomic actions are only used as building blocks for basic and collaborative actions.

Action prerequisites (humanActionPrerequisite and robotActionPrerequisite) are used to describe dependencies to actions of human and robot, which are required to have been executed directly before executing the given action. These prerequisites can be provided for collaborative and basic actions. In this way, dependencies like “Pick Object needs to be executed by an actor before being able to place the same object” or “An actor can only pick an object, if it did not pick something else right before,” can be described. For a given action type, a union of prerequisite actions can be defined, i.e., different valid action prerequisites can be defined for one action at once. Consider for example, the user has already picked an object. Logically, the user can then place the object down on the table or keep holding it. It is not physically plausible to pick another object while already holding onto an object. The system models these relations as prerequisites and thereby, the basic actions are given in Table 4.

**Table 4:** Action prerequisites-human and robot basic actions.

**Human and robots basic events**			
Idle			
Pick and Place	Pick and Insert	Pick and Hold	
Idle	Idle	Place	Insert
		Idle	Idle

Similarly, the prerequisites to follow when proposing collaborative actions are given in Table 5.

**Table 5:** Action prerequisites to consider for collaborative actions.

** Human events**	** Robot events**
Pick and hold	Pick and hold
**COLLABORATIVE EVENT MOUNT**	
Idle	Place
	Idle
Idle	Pick and hold
**COLLABORATIVE EVENT PICK AND MOUNT**	
Idle	Place
	Idle
Idle	Pick and Hold
**COLLABORATIVE EVENT HANDOVER AND INSERT**	
Idle	Place
	Idle
Idle	Pick and Hold
**COLLABORATIVE EVENT HANDOVER AND PLACE**	
Idle	Place
	Idle

Considering Tables 4, 5, the number of possible actions in the initial state are now reduced to 9 actions for the user (consider Table 4, human is idle, hence the user can perform three actions and by considering the object affordances, the user can manipulate 3 objects in the initial state) and 9 actions for the robot. Given these reduced set of actions based on the user selection, the corresponding action is stored for that state. This is done with the help of interactive learning and is explained in the next section.

### Reinforcement learning

Reinforcement Learning (RL) is an area of machine learning that defines a class of algorithms that enable a robot to learn from its experience. In this work, the standard notation of Markov Decision Process (MDP) is used to define Reinforcement learning. In a MDP, any state *s*_*t*+1_ occupied by the robot is defined as *s*_*t*+1_ = *f* (*s*_*t*_, *a*_*t*_), where *s*_*t*_ is the previous state and *a*_*t*_ is the action taken in state *s*_*t*_. The 5-tuple < *S, A, P, R*, γ >, define the MDP, where S is the set of possible world states, and *A* denotes the set of actions available to the agent in each state. The probability function *P*: *S* × *A*→*Pr*[*S*], describes the transition probability of State *s*_*t*_ to State *s*_*t*+1_, when an action *a*_*t*_ is performed on State *s*_*t*_. The reward function is defined as *R*: *S* × *A*→*R*, and γ denotes the discount factor (0 ≤ γ ≤ 1). *P* and *R* together describe the dynamics of the system. The goal of a RL algorithm is to find an optimal policy π by approximating the function *Q*:*S* × *A*→*R*. Here, *Q* maps the state-action pairs to the expected reward. The optimal policy *n*: *S*→*A* maximizes the expected reward. The optimal policy, π defines the best possible action to perform in a given state with the goal of obtaining the maximum reward.

In this work, the learning algorithm is based on Q-learning RL (Watkins and Dayan, 1992), With the successful application of IRL in Thomaz et al. (2006) and Suay and Chernova (2011) as inspiration, we extend IRL to be used in learning a complex human robot collaborative assembly process as shown in Figure 1.

For generating the options of different actions that can be chosen/executed at a certain process state, the following assumption holds: Each learning process starts at the Initial State of an Assembly Process (e.g., all objects present in work environment, the actors are idle).

- Given the description of the Initial State (see semantic model, earlier), and the knowledge about the object classes of interest for the given use case a list of objects of interest for the given (initial) process state is generated (from the knowledge of the process constraints).
- Consider actions that were executed previously (in initial PS, nothing was executed before): If the last actions include “Hold” atomic actions, the effects of these previous actions are computed based on defined rules (e.g., Object X is hold by actor A and not visible in the work space, but can participate in a future action).
- Based on the defined action prerequisites, a rule is executed to find all actions, performable by the actor type of interest, which can be executed by considering only action prerequisites.
- For each possible action type and considering the objects available and hold by actors, a rule checks for possible configurations of the given objects and the actor of interest.
- Finally, especially for collaborative actions like “PickAndMount,” “Mount,” “Handover…,” additional constraints of the given objectOfInterests for an action and previously hold objects need to be checked. For example in this last step, it is verified e.g., if an object of interest can be really combined with a previously hold object to build a compound object.

The rules are partly implemented in an action type specific fashion. Through the concept of unification in Prolog, and appropriate constraint-checking rule e.g., for the given action class, can be determined clearly. The output of the main prolog rule is as follows: For a given Process-State and the actor(s) of interest (e.g., human, robot, or collaborative i.e., both actors), a list of possible actions is returned. Moreover, a list of lists containing possible objects of interest for each of these actions is calculated. Lastly, a list containing a lists of lists is returned, which contains possible combinable targets objects for the given action and the possible objects of interests. Using this information, a chosen action to be executed in a given process state, can be clearly parameterized.

#### Learning phase

For the learning process which is based on the Q-learning, the description above mention the generation of state-action pairs in the assembly process. In order to learn an optimal policy the system can either explore or exploit. The strategy to explore and then observe a reward can be very time consuming, as it require huge amount of trials to find the optimal policy. The strategy followed in this work makes use of the human operator to exploit the users presence to find an optimal policy. In the initial state, given the state-action space, the system proposes a set of solutions to the user and initializes the Q table (state-action pair rewards) with zero. The system uses the criterion mentioned above to propose a set of plausible state-action pairs to the user. The user then selects the best possible option (according to the user's choice). This state-action pair is then recorded and is stored with the highest Q value possible. Thus, for the initial state, the chosen action is performed and the Q value is stored. The resulting state (as a consequence of performance of the chosen action) is now the next state to the system. The system observes (perception reasoning) the resulting state and proposes a new sets of state-action pairs. The human user then selects another suitable action for that state, the system stores the highest Q value for the state-action pair, performs the action accordingly and proceeds to the next state. In this fashion, the system proposes a set of state-action pairs to the user in each state, the user selects a suitable action, the system records the highest Q value for that state and performs it and proceeds to the next state. After reaching the final state (specified by the user), the Q table with the optimal policy is already achieved with the help of user interaction and the proactive proposal of the system.

#### Online phase

During the online phase, the system loads in the learned policy, observes the current environment and checks if the current state has an optimal action already learned during the learning phase. The system proceeds with a suitable action if an optimal action policy exists for that state observed. This is continued until the assembly is completed. If in case, the action executed results in a state not known to the system, a deviation is triggered and a new policy learning option is given to the user Akkaladevi et al. (2017b).

## Experimental setup and the learning approach in practice

**Figure 6:**
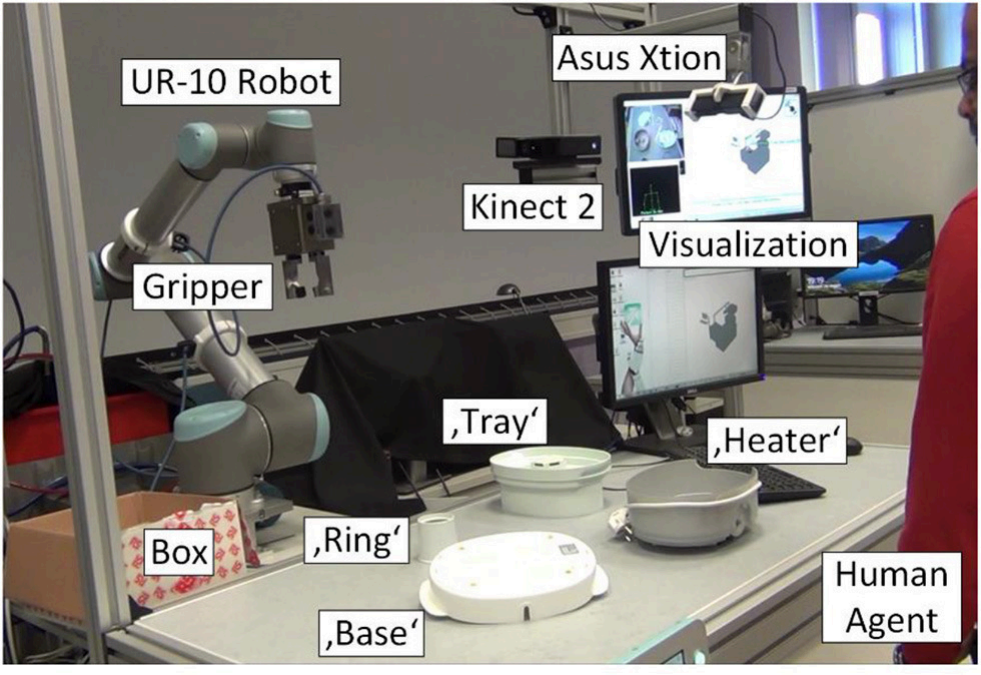
Experimental setup including robotic manipulator, 3D sensor system, Human Machine interactive visualization, objects and human agent (Akkaladevi et al., 2017b).

The experimental setup for the HRC assembly process scenario is depicted in Figure 6. The objects (base, heater, tray ring) located in the workspace are related to the chosen assembly process as described in Table 1 for the evaluation. The setup consists of a UR-10 robotic manipulator (Universal Robots, 2016) with 6 degrees of freedom, which is equipped with a SCHUNK electric parallel gripper. Two RGB-D sensors, Kinect 2 and Asus Xtion Pro provide depth data to the perception system, to enable human action recognition as well as object localization and tracking.

**Figure 7:**
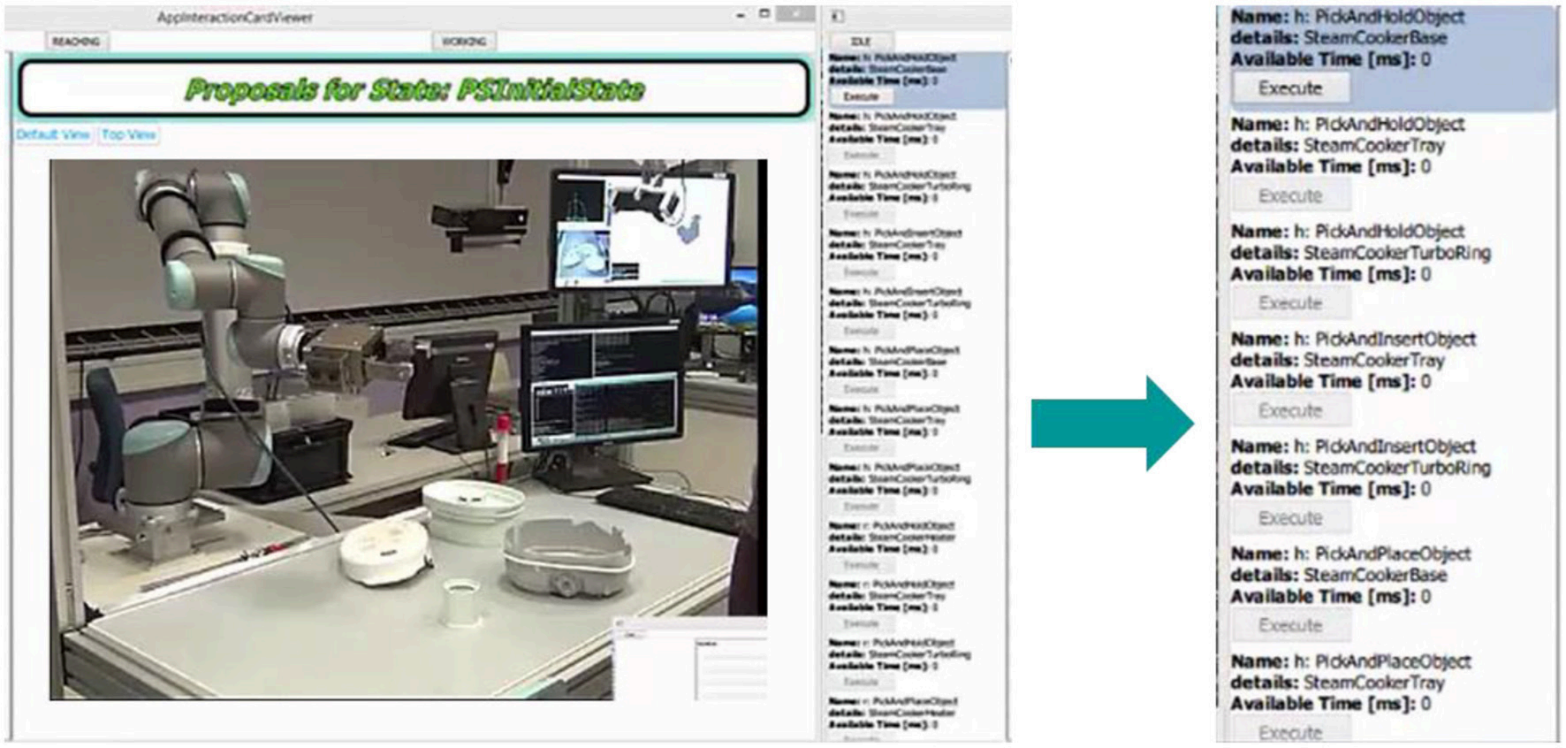
The possible set of actions in the initial state of the assembly process.

Consider the initial case of the assembly process, as shown in Figure 7. There are 4 objects (H, B, T, R) on the table and the user and the robot are in idle state. The robotic system proposes a set of possible actions as described in section Learning Methodology. The system considers the user and robot capabilities, object affordances, the action hierarchy and prerequisites before generating a final set of possible actions. Initially there are about 18 choices available for the user to decide between the type of action (and on which object) and also choose the agent (human or robot) that will perform the action.

**Figure 8:**
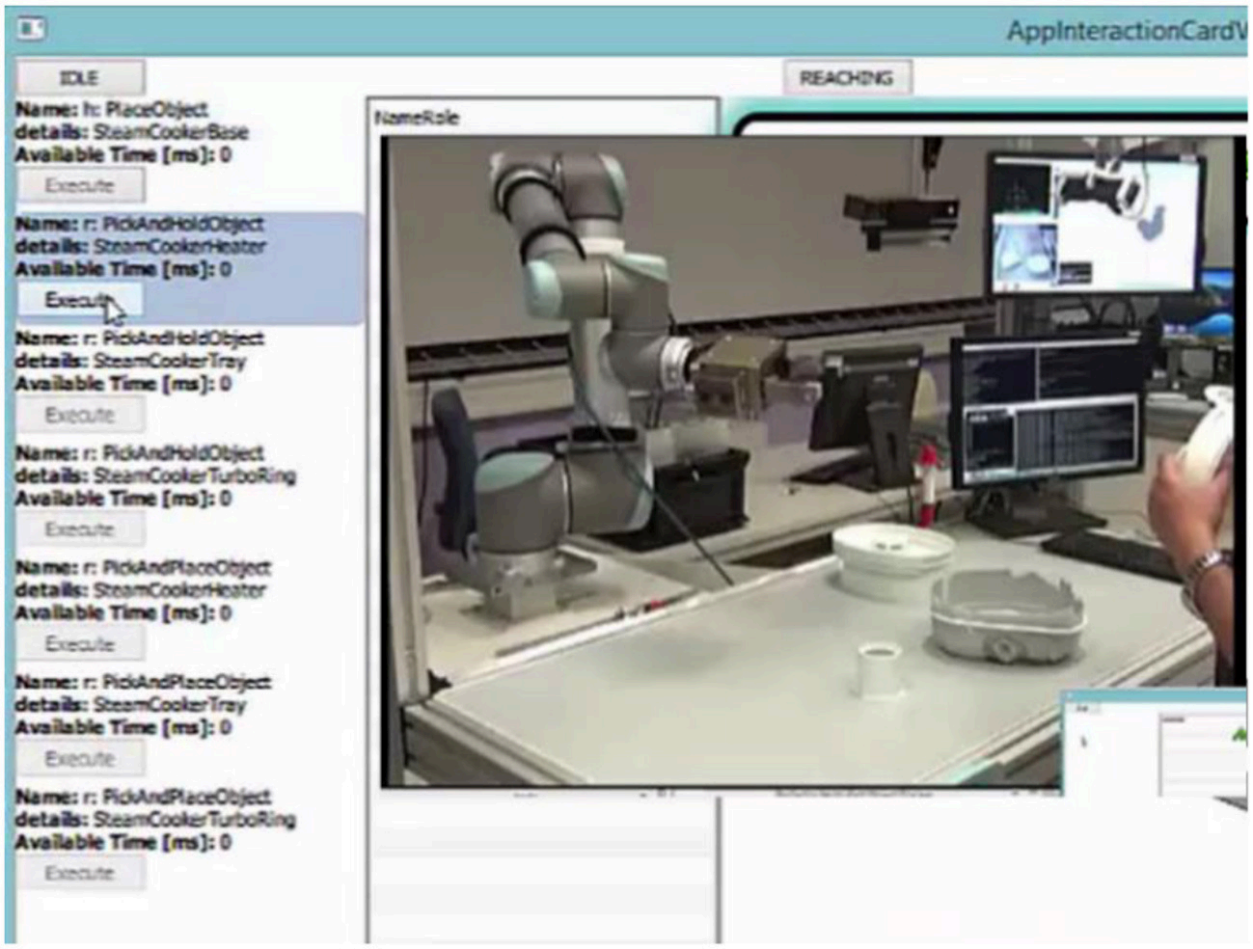
The possible actions after user has picked up the base object.

Let the user select an action (human to pick and hold base), the system executes (in this case waits until the user pick and holds the base object–this is detected using the event detector as described in section KoMoCog Architecture). After executing the action, the system records the state and then suggest the next set of actions as shown in Figure 8. It should be noted that the possible actions are now drastically reduced to just 7 actions. The reason is since the user is already holding the base object, the number of actions for the user is limited and the same applies for the robot (according to Tables 3, 4).

**Figure 9:**
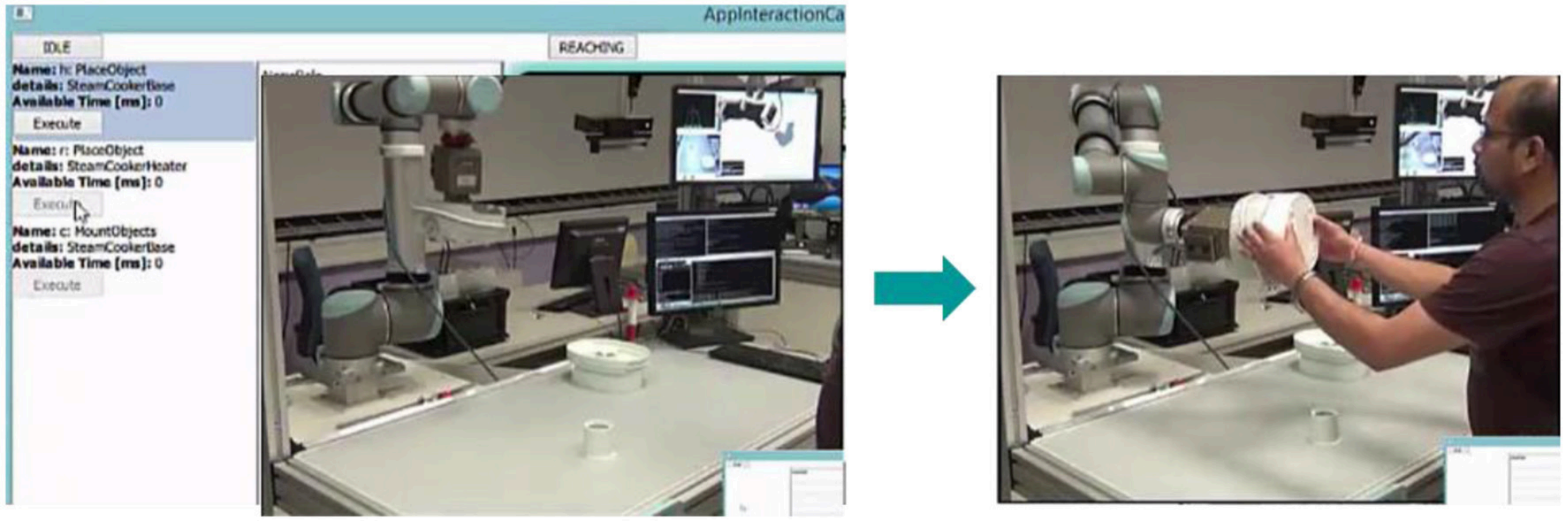
Robot proposes all possible basic and collaborative actions; User chooses collaborative actions and performs it in coordination with the robot.

Given the reduced set of actions, now the user selects the robot to pick and hold the heater. The robot executes the actions and now proposed a set of new actions. Note: until this point, only basic actions were proposed since the action prerequisite for collaborative actions were not satisfied. After the robot picks and hold the heater, it realizes that now even collaborative actions are possible and hence it proposes all basic actions and collaborative actions possible as shown in Figure 9.

Considering Figure 8, the number of possible actions proposed are about 9 each for the human and the robot in the initial state. When only using agent capabilities and object affordances as mentioned in Table 2, where the number of possible actions are 33. While also considering action hierarchy and prerequisites, the number of options are reduced further as shown in Figure 8. The use of action prerequisite is more evident in Figure 9, where the possibilities are further reduced to 7 and 3, respectively. This way of teaching a robot an assembly process also has an additional advantage that different users can teach different ways of doing the assembly process. The system is capable of remembering different sequences of doing the assembly process, where an episode (a complete assembly process) taught by a user can be stored according to the user profile. This way, user preferences can be taken care of while performing the assembly process during runtime.

## Evaluation

For Human Robot Collaboration (HRC) to be possible, the robotic system should first understand the circumstances under which it operates and then act, adapt and react accordingly. However, with the presence of the human at the center of its unpredictable nature, efficient HRC is quite difficult to achieve. Recent studies on cognitive architectures (Bauer et al., 2008; Pedersen and Krüger, 2015; Steinmetz and Weitschat, 2016; Stenmark and Topp, 2016) for HRC suggest that for an efficient HRC, the robotic system should semantically create a link between its observations of the environment and the action chosen for execution in a close loop. This allows the robotic system to choose a suitable action, execute, verify and adapt accordingly. The idea of these approaches involve representing the knowledge about the environment in the HRC using some form of semantic rules (ontological web description language, description logic, first order logic). Once defined, the robotic system utilizes its observation capabilities (perception) and queries the knowledge base to infer and reason about the current state of the environment and choose an action accordingly.

However, modeling the knowledge using OWL is a tedious process and the complexity increases with the complexity of the assembly process involved. We argue that the proposed learning framework allows for ease of modeling the semantic knowledge in the assembly process. To evaluate this learning framework, the use case chosen as shown in Figure 6, includes 4 objects and involves 33 actions. The number of states possible result in 132 states. The number of states is a result of a number of combination of states possible with the given set of objects and the action possibilities. In such a huge state-action space that involve dynamic human robot collaboration, modeling the knowledge becomes very tedious as manually creating semantic link is both time consuming and also has to be updated again manually.

To reduce such a complex state space, the learning framework considers the state-space reduction strategy mentioned in Tables 3–5. The state space is further reduced by considering human interaction and considering the objects of interest in that interaction as shown in the video link.

The video link^1^ shows the complete learning framework in action. The video shows the individual modules and the object involved are demonstrates the intended use case. This is followed by the learning framework, the interaction with the human operator and the process involved. The video also demonstrates the capability of the learning process to deal with deviations. This deviation handling (Akkaladevi et al., 2017b) is not the focus of the work presented in this paper and hence is not explained in detail. The second part of the video showcases the applicability of such a learning framework and compares two different cognitive architecture. These two cognitive architectures have the same modules. However, they differentiate each other in the sense that one exploits the semantic links learned through the proposed framework and the other, which does not. The evaluation highlights the obvious advantage of such use. The framework not only learns the sequence of the assembly process but also learns the semantic link between each step of the assembly process. Exploiting the semantic link to understand the environment, choosing a suitable action and verifying an executed action is of vital importance to complete an HRC assembly. This can be only possible if the system is aware of the changes in the environment, is able to reason, plan and execute all of which is made possible by the use of the learning framework.

## Conclusion

Conventionally, robotic systems were used in highly automated production scenarios, where robots were installed behind closed fences and were used to perform repetitive tasks. With the recent developments in collaborative robotics are allowing these robotic systems to break the fences and move toward working hand in hand with the human users. These developments are in line with recent demographic changes in productions scenarios from mass production to mass customization, where reconfigurable robotic systems will play a key role. Programming such re-configurable robotic system in short amount of time is of vital importance to manage variations of production processes. In this work, we presented such a learning framework, where the robotic system is capable of learning simple tasks (using task based formalism) and combine these simple tasks in an intelligent fashion to learn complicated assembly processes.

In this learning framework, the robotic system with the prior knowledge of simple tasks suggests the user a set of possible actions, when presented with a set of objects. These proposals take into consideration the agent capabilities, object affordances and action hierarchy and prerequisites to reduce the number of proposal provided to the user. This allows the user to take part in the learning process and also enables learning of multiple assembly processes. The next steps in this work would be to perform detailed evaluations and user studies of the learning framework considering a number of users (experts and non-experts). The evaluation criteria would be mainly interested in the time taken, ease of use and intuitiveness of the learning framework.

## Author contributions

SA and MP conceived the presented idea, developed the theory and performed the computations. SM carried out the experimental evaluation. SA wrote the manuscript with support from MP, SM, CE, AP, and BR.

### Conflict of interest statement

The authors declare that the research was conducted in the absence of any commercial or financial relationships that could be construed as a potential conflict of interest.

## Footnotes

^1^Video: https://youtu.be/hIsWTUllu3g
